# Moomins Take the Floor. Finnish Trolls in Contemporary Mass Social (Media) Events

**DOI:** 10.1007/s10583-022-09497-6

**Published:** 2022-08-11

**Authors:** Hanna Dymel-Trzebiatowska

**Affiliations:** Institute of Scandinavian and Finnish Studies, University of Gdańsk, Gdańsk, Poland

**Keywords:** Moomins, Tove Jansson, #OURSEA, Invisible child campaign, Polish abortion strikes, Literary references

## Abstract

Tove Jansson (1914–2001) was an outstandingly talented Finnish-Swedish artist, recognized worldwide mostly as the creator of the Moomins. Although the last of the nine-volume series (1945–1970) about the internationally popular Finnish trolls, *Moominvalley in November*, was published over fifty years ago, the Moomins’ and Jansson’s popularity is still rising, which testifies to the universality and timelessness of the books as well as the thoughtful marketing strategies of Moomin Characters Ltd, the company responsible for copyright supervision. In this article I discuss the Moomin characters’ representation in three events set in contemporary (2017–2021), extratextual societal contexts, both strategically planned and co-organized by Moomin Characters Ltd and spontaneously organized by ordinary people. The motifs and figures from Moominvalley which have been applied in (1) the Invisible Child campaign, (2) the #OURSEA campaign, and/ (3) the women’s strike protests in Poland are exemplified and juxtaposed with their literary images in the nine Moomin books by Jansson. My conclusion is that the references are based on a selective reading and focus on beneficial—in specific contexts—facets. On the one hand, it proves the Moomin books’ versatility and topicality, while on the other, it is not inclusive and disregards the holistic message of the saga, which is an evolving continuum. Furthermore, this way of reading reinforces a single address of the unequivocally double-addressed series.

## The Genesis

Tove Jansson, an illustrator and painter, started to write her first Moomin book, *The Moomins and the Great Flood*, during the Winter War in 1939 to distract from the despair and fear, to escape the reality, and to shape her own private catharsis—an original, fairy-tale-like purgation of negative emotions. Six years later, in 1945, she decided to publish this book, which when reprinted in 1991 appeared with her personal preface—interestingly, the only one within the whole series: “One’s work stood still; it felt completely pointless to try to create pictures. Perhaps it was understandable that I suddenly felt an urge to write down something that was to begin with ‘Once upon a time.’ ” (Jansson 2018/1945, p. 9). The appearance of the novel was hardly noticed on the book market both in Finland and Sweden, shadowed by Pippi Longstocking’s debut, and nothing really hinted that one day the Moomins might compete with the redhaired tomboy for the number one position in Nordic children’s literature. But *The Moomins and the Great Flood* was shortly followed by *Comet in Moominland* (1946) and *The Hobgoblin’s Hat* (1948), and the situation was to alter. As Westin notes, “In *The Hobgoblin’s Hat* Tove Jansson the writer enlarged her Moomin world, sharpened her aesthetic, honed her vocabulary and anchored the codes of her fictive world to a firm foundation. She worked on the personalities of her characters, refining their characteristics and use of language” (Westin, 2014/2007, p. 157).

While developing the fictional world of the Moominvalley, Jansson, a brilliant observer, drew on the images of her own family, friends and reality, and simultaneously, in a still escapist manner, structured a verbal and visual vision of a dreamlike community and a better future. Although her books have been traditionally labelled and marketed as children’s literature, already the first volumes were evidently double-addressed (Dymel-Trzebiatowska, 2016, 2020) as regards both a sophisticated sense of humour and philosophical references. These assets were recognized by readers and reviewers, and *The* *Hobgoblin’s Hat* (a literal translation of the original, Swedish title *Trollkarlens hatt*) made not only a national but also an international breakthrough after the book was translated into English in 1950 as *Finn Family Moomintroll.* The volume was followed by six other Moomin volumes^1^ and concluded with *Moominvalley in November* in 1970.

Over the two and a half decades Jansson gained the status of a star author and Moominmamma, largely due to the publication of comic strips in *Evening News*, launched in 1954. The *Evening News* was a big daily paper in UK, and at the height of the success her strips were translated and read in 40 countries. Regrettably, the fame and growing assignments turned craving for writing and drawing into a stressful burden and, in 1961, made the exhausted artist call the trolls “happy idiots” (Westin, 2014/2007, p. 262)—presumably a tough label to accept for many Moomin fans. Nevertheless, Jansson’s life was radically changed by the Moomins and in particular by the Moomin business which got underway in the 1950s. The merchandise seemed to take endless forms, and at the outset it was Jansson who supervised all the projects. As Westin reports:

The whole repertoire was controlled by Tove herself, whose exhaustive lists document a growing and increasingly motley supply. There were fine wallpapers stamped with Moomin figures, printed textiles and pictures for children’s rooms, crockery services, wrapping paper, cards, writing papers, calendars and diaries, puzzles, bookmarks, braces and suspenders, ties, mugs and even hairbands. The production of these objects stretched like a serial from file to file. One such file is divided into sections marked ‘plastic’, ‘ceramic’ and ‘Moomin dolls and cloth’. (2014/2007, p. 228)

When the Moomin business was expanding and killing Jansson’s free creativity, in 1958 she and her brother Lars decided to set up a company which in 1979 was transformed into Moomin Characters Ltd, to this day run by family members, with Sophia Jansson, Tove’s niece, as Chairman of the Board and Creative Director.

## The Never-Ending Boom?

Another input into the Moomins’ international career came surprisingly from the other side of the globe, when in the early 1990s the Japanese-produced cartoons fuelled the Moomin business again and generated the second wave of the boom. The filming was preceded by the success of the books:

The Japanese publishers saw the books as a response to an increasing need for togetherness at a time when family life was changing. Families were breaking up and their members moving off in different directions. The Moomin books presented a family which gave every member freedom yet at the same time held them together, a positive combination of an older era and a new one. (Westin, 2014/2007, p. 343)

In 1995, on the wings of success, the managing director of Moomin Characters Ltd announced that they were aiming “to establish the Moomins alongside Donald Duck and Asterix” (Tarkka and Marten, 2020), and while following the firm’s constant intensification of marketing, these words were not an empty promise. Today the company’s agenda is multifarious and covers many domains: (1) Tove Jansson’s books and life work, (2) Tove Jansson as a writer and a visual artist, (3) Moomin-related products, and (4) campaigns—all advertised and regularly updated on the official website, Facebook pages, Instagram, YouTube channel and through newsletters. A representative example of the first category is the collectors’ editions of Moomin books in vintage layouts, of the second—current news on the successful biopic *Tove* (2020), of the third—the spectacular Moominvalley Park that opened in Tokyo in 2019 and its current exhibitions, 3D animation series (2019) or original seasonal mugs by Arabia, and of the fourth—the Invisible Child campaign (2017) and #OURSEA (2020) or the initiative launched in February 2021—Reading, Writing and the Moomins. Furthermore, another intriguing form of expansion is promoting “Moominous” values such as love, courage and equality, or a Moomin way of life, which seems to be creating an alternative to Finnish *sisu*. This seems to be building  a popular form of Nordic country branding with national, hypothetically existential concepts, like Danish *hygge* or Swedish *lagom*.

It is noteworthy that the Moomins are not restricted to a fictional domain of books, comic strips or spinoffs but conspicuously transcend it, entering and interacting with the real world. In line with the tips on the website, you can “moominize” your day both online and offline by, for example, getting inspired by Tove Jansson and reading *Letters from Tove* (Moomin, 2021a). The intensification of the promoted online activities is of particular interest as it proves the flexibility and quick adjustment to the conditions imposed by the coronavirus pandemic, which also manifests itself in the production of face masks with Moomin characters by Aurora Decorari or promoting Moomin exhibitions in Sweden in July 2020 as a form of “Moominous” staycation.

Interestingly, the 77-year-old Moomintroll gave way to neither Harry Potter nor the Wimpy Kid. On the one hand, the maintenance or even growth of the Moomins’ international popularity twenty years after Tove Jansson’s passing results from the long-term, systematic work of Moomin Characters Ltd. On the other hand, the Moomins became globally associated with Finland and recognized as one of the most typical Finnish items, promoted by the country itself. This small (in terms of population size) nation is commonly famous for its high-quality education, mobile phones, minimalist yet cosy design, and … the Moomins. And although Finland enjoys one of the best nation brands worldwide, it is still developing it, employing Moominvalley and its original dwellers.

The country brand webpage thisisFINLAND (finland.fi), produced by the Ministry for Foreign Affairs of Finland and published by the Finland Promotion Board, includes extensive coverage related to the Moomins. The contributions overview the history of the book series and their creator, Tove Jansson, inform about new initiatives by Moomin Characters Ltd or refer to anniversaries such as the Moomin’s 75th birthday celebrated in 2020. Symptomatically, they emphasize, often in the headlines, both the importance of the figures and their national affiliation, labelling them “Finland’s Moomins”, “classics”, “semiofficial national symbols” or “a national treasure”.

Although Moomin Characters works on commercial premises, as Sophia Jansson pointed out for *thisisFinland*, there is also room for “exhibitions or activities on a nonprofit basis” (Marten, 2010). The campaigns launched and promoted by the company relate to Tove Jansson’s life and her books, and create an intimate bond between the artist, her lifework and the urgent problems of the present, indicating their topicality.

## The Invisible, Tove and Feminism

In 2017 Moomin Characters Ltd and Oxfam—an independent, UK-based charitable organization focused on the alleviation of poverty and injustice—joined forces in a campaign whose primary goal was to empower girls and women around the world. The event was broadly promoted and applied a short story, “The Invisible Child” from *Tales from Moominvalley*, published originally in Finland in 1962, as a pivot. The official Moomin website announced it in a post on 26 September 2017, presenting the campaign’s character and goal: “The partnership centres around *The Invisible Child*, a short story by Moomin creator Tove Jansson, which will be available to buy from Waterstones, the Moomin Shop, Covent Garden and Oxfam shops, with at least £4 from each sale being donated to Oxfam’s women’s projects worldwide” (Moomin, 2017).

Except for a special, collectable edition of “The Invisible Child” paired with the short story “The Fir Tree” and reinforced with the cover slogan “Share the gift of the Moomins”, Oxfam has been selling a range of other Moomin-related products, including stationery, handkerchiefs, tote bags and kitchenware. Other partners,  such as Fiskars and Finlayson from Finland or Macmillan Children’s Books, supported the campaign by contributing part of the revenue from their Moomin products. In June 2018, a new attraction was launched when a famous British actor, Bill Nighy, recorded his reading of the short story. Overall, the campaign has been very successful, since by 30 December 2020 Moomin Characters officially reported that their partnership with Oxfam had raised 1 million pounds for charity projects supporting women and girls around the world.

The above-mentioned first post from the Moomin news webpage highlights a relation between Jansson’s text and the initiative’s aims:

*The Invisible Child* is about a little girl who turns invisible after being badly treated by the woman supposedly caring for her. She is given a place to stay at the Moominhouse and, when shown warmth, kindness and respect by the Moomin family, she gradually reappears and regains her place in the world—a right that every woman and girl should have. (Moomin, 2017)

Further, there are references to Tove Jansson herself when Sophia Jansson points out her aunt’s brave attitude and relates it to a long tradition of equality in her home country:

Tove was a strong and independent woman who lived life the way she wanted to—unlimited by ideas about how a woman should behave or what her role should be— which isn’t too surprising considering where she grew up. Finland has always been a leader in women’s rights and was the first European country to give women the vote in 1906. However, not everyone is this fortunate, and I’m sure that Tove would be very glad that her stories are going to help women all across the world escape poverty and find their voices. (Moomin, 2017) indent this para? 

On 26 September 2017 the Invisible Child event was also presented and advertised in *The Guardian*, in the article “The Moomins: Tove Jansson’s feminist legacy” focusing attention on feminism, pinpointed by Sophia Jansson’s strong argument: “I’m not sure Tove ever used the word ‘feminist’, but she was one in every sense of the word” (Dening, 2017). Interestingly, the author, Lizzy Dening, starts with the observation that post-war stories about the Moomins might not be the first place we would look for feminist icons, although with the article’s title she sends an opposite signal. The solution to this apparent dilemma is provided in the observation that “the more you discover about the women in Moomins creator Tove Jansson’s life, the more it makes sense” (Dening, 2017), and I do agree with her. However, only a very insightful, comprehensive and experienced reading both of Jansson’s biography and her books can lead to this conclusion. Let us take a quick look at some female characters of the saga, including Ninny, a pivotal figure of the Invisible Child campaign and an eponymous figure of the short story. The girl is merely one of numerous figures in Moominvalley and certainly not the most recognizable one. The image of Moominvalley females is shaped by the predominant, considering the quantitative and qualitative occurrence, character—Moominmamma.

Moominmamma’s attributes are a striped apron and a black handbag, which symbolize her protectiveness, resourcefulness, foresight, and basically her huge concern about others. She is also kind, helpful, delicate, modest, altruistic, yielding and intuitive, and thus represents a literary figure characterized by all features which according to cultural feminists are traditionally associated with women (Putnam Tong, 2002, p. 173). Paradoxically, with this package of characteristics she is often interpreted by readers as an ideal mother. Modelled after Signe Hammarsten-Jansson, Tove Jansson’s own mother, she always humours Moominpappa and can be viewed as subject to masculine domination, a popular concept by Pierre Bourdieu to approach gender inequality. This form of symbolic violence involves by definition acceptance of the subordinate, in this case manifested across the domain of gender (Bourdieu and Wacquant, 1992). Moominmamma accepts her husband’s moods and whims, constantly suggesting solutions so ingeniously that he takes them for his own. But it is the androcentric thinking that dominates the family, and most decisions are taken to satisfy the male ego. Significantly, being the perfect Moominmamma, she became one of the most beloved and admired figures, a foundation of the ideal, happy family, which contributed to the popularity of the Moomins (Dymel-Trzebiatowska, 2016, p. 72).

Snork Maiden is also depicted as stereotypically girly by, for example, being recurrently rescued by Moomintroll playing a Tarzan role, and even if adults can read it as a parody of gender-coded ideals (Westin, 2014/2007, p. 144), for novice readers it could be a reinforcement of gender stereotypes^2^. Furthermore, Snork Maiden is rendered as focused on her looks, and her attribute—a glossy anklet—emphasizes it suggestively. Fillyjonk is another type of stereotyped woman fixed on cleaning and raising well-behaved children. She is also neurotic and feels oppressed by her own mental constraints.^3^

It can be stated that this is a common vision of the Moominvalley based on a selective and superficial reading of the saga, whereas a more profound and holistic approach reveals a shadow text (Nodelman, 2008) where both Moominmamma and Snork Maiden act as empowered women and prove their superiority over male figures (Westin, 2014/2007, p. 145). It is true, but still a majority of the scenes with their participation, and a simple quantitative criterium, largely fortify the female-coded behaviours.

On the other hand, other females, such as Too-ticky or Little My, serve as a counterbalance since they contradict the gender stereotypes and demonstrate that being a woman does not necessarily involve cooking, cleaning and being attractive for men. Too-ticky, whose prototype is Jansson’s partner, Tuulikki Pietilä, is featured in *Moominland Midwinter* as the embodiment of wisdom and security. She is befriended by winter and enables Moomintroll to survive it, sharing philosophical observations. Little My appears in the fourth volume, *Moominpappa’s Exploits*, and her characterization crystalizes in *Moominsummer Madness.* From then on she is becomes a manifestation of—inversely proportional to her tiny size—strong will, independence and assertiveness. Even if she acts egoistically and her remarks can seem mean, she is still hilarious due to her diminutiveness, her frowning face in the illustrations and her black humour. If we were to find a feminist in the Moominvalley, she would be the best candidate. In sum, there is no single female image in the Moominvalley as there is no single female image in reality; they are diverse, and or, it is up to readers to spot the irony at work in the depiction of non-normative and normative gender roles...?

Coming back to Ninny, she was brought one autumn evening to the Moomin family by Too-ticky. Ninny was invisible and, as Too-ticky explained, her unusual condition was caused by her curator’s irony. The storyline was influenced by Karen Horney’s book *Neurosis and Human Growth: The Struggle toward Self-Realisation* (1950), which Jansson read and appreciated (Westin, 2014/2007, p. 282). The Swiss psychoanalyst conceptualized three neurotic solutions—self-effacing, expansive and withdrawing—and Ninny, in short, represents the first one. A great frustration impeded the development of her genuine inner self and furthered her self-alienation, which in Jansson’s text and image was conveyed by her physical invisibility.

The girl, like all guests, was welcomed into the Moomin family and treated with equality and respect. Although at the outset she typified a rigidly obedient compliance, at the end she could regain her visibility thanks to an old recipe labelled by Moominmamma’s nanny, “If people start getting misty and difficult to see” (Jansson, Tove. (2019/1962). The Invisible Child. In Tove Jansson, Tales from Moominvalley (pp.115–135). Transl. Thomas Warbuton. London: A Puffin Books p. 122). Not without significance for the treatment was Little My’s advice: “‘She can’t get angry,’ Little My said. ‘That’s what’s wrong with her. Listen you,’ My continued and went close to Ninny with a menacing look. ‘You will never have a face of your own until you’ve learned to fight. Believe me’” (Jansson, Tove. (2019/1962). The Invisible Child. In Tove Jansson, Tales from Moominvalley (pp.115–135). Transl. Thomas Warbuton. London: A Puffin Books. 2019/1962, p. 128). In the final scene, Moominpappa while pretending to push Moominmamma into the sea gave the girl an opportunity of accomplishing the recovery:

Ninny had sunk her small invisible teeth in Moominpappa’s tail, and they were sharp.

‘Good work!’ cried My. ‘I couldn’t have done it better myself!

Ninny was standing on the landing stage. She had a small, snub-nosed, angry face and a red tangle of hair. She was hissing at Moominpappa like a cat (Jansson, Tove. (2019/1962). The Invisible Child. In Tove Jansson, Tales from Moominvalley (pp.115–135). Transl. Thomas Warbuton. London: A Puffin Books 2019/1962, p. 133).

After the publication of *Tales from Moominvalley*, the book was assessed as filled with psychological sophistication and “the reviewers wrote of Tove Jansson as a therapist, moralist and educationalist” (Westin, 2014/2007, p. 281).

But Ninny was not the only literary character in the Moomin suite representing the invisible. As Westin points out, in the 1960s Jansson’s artistic works had a common denominator, both on canvas and in writing, “the idea of the invisible” (2014/2007, p. 279). Focusing only on literary characters, we might mention the mice who inhabited and served at Too-ticky’s place in *Moominland Midwinter*. “They’re all so shy that they’ve become invisible” (Jansson 2017/1957, p. 32), explained the hostess to the visiting Moomintroll. In the same book there are *the others* representing the same category, again presented by Too-ticky: “There are such a lot of things that have no place in summer and autumn and spring. Everything that is a little shy and a little rum. Some kind of night animals and people that don’t fit in with other and that nobody really believes in” (Jansson, 2017/1957, p. 52). Further representatives of the invisible are certainly Salome from the same winter Moomin book, a nameless creep from the short story “The Spring Tune”, a protagonist of the picturebook *Who will comfort Toffle?* or Toft from *Moominvalley in November*. Holistically viewed, they constitute a substantial set of diverse figures, living on the fringes, who have been given voice by Jansson. As she clearly conveys, there are numerous variants of invisibility, which is genderless, ageless and species-less.

## Moomins, Tove and the Sea

One of the focal points of the Moomins’ 75th birthday celebrations in 2020 was unquestionably the #OURSEA campaign launched by Moomin Characters Ltd in cooperation with the John Nurminen Foundation (oursea, 2021). Its goal is to raise funds to save the Baltic Sea and its unique cultural heritage, and the organizers highlight that the donation of €10 can remove 40 kg of blue-green algae.

The official website of Moomin Characters Ltd has advertised #OURSEA regularly and devoted a large space to it on its official website, including frequent, regularly upgraded, posts. They are of different character and focus on money collected so far and the purposes it has already been partly spent on, related exhibitions and initiatives benefitting the campaign, #OURSEA-themed merchandise, Tove Jansson’s relations with the sea, and the importance of the maritime motifs in the Moomin books, which is of special interest for my study.

When on 14 January 2020 the first post on the Moomin website announced the campaign’s launch, it drew on, (1) a quotation from *Moominpappa at Sea*, (2) the significance of the sea in Jansson’s private life, enhanced by her two black and white photographs, and (3) the fact that this beloved sea is today affected by dangerous eutrophication. The simple syllogism led to the conclusion that Moomin fans should consider donation and “save the Baltic Sea and its cultural heritage for future generations” (Moomin, 2020a). Further posts developed the formula of supporters’ potential reactions: “To take part in the campaign you can make a direct donation via www.oursea.fi, buy a campaign product, learn more about the sea, the challenges it faces, and solutions needed to help improve the situation, as well as influence your friends, family, colleagues and politicians to take action” (Moomin, 2020b). The options of ecological behaviour appeared really ingenious and website readers were in the following months encouraged to eat local fish with reference to a fishing motif in Moomin books, choose green electricity which is related to Hattifatteners as a natural source of energy, or wear old trousers and thereby follow Snufkin’s environmentally friendly lifestyle.

Focusing on the references between the literature and the campaign, the aforementioned citation used initially in January 2020 was an extract from the last scene of *Moominpappa at Sea*:

He came to the edge of the water and stood watching the breakers. There was the sea—his sea—going past, wave after wave, foaming recklessly, raging furiously, but, somehow, tranquil at the same time. All Moominpappa’s thoughts and speculations vanished. He felt completely alive from the tips of his ears to the tip of his tail. This was a moment to live to the full (Jansson, Tove. (2019/1965). Moominpappa at Sea. Transl. Kingsley Hart. London: A Puffin Books. 2019/1965, p. 259).

Since the book practically says goodbye to the Moomin family, which is absent in the next and last, ninth volume, it seems of particular significance. Furthermore, as Westin stressed, “*Moominpappa at Sea* is the real breaking point between Tove’s writing for children and writing for adults, a coming to terms with her narrative of the Moomin world, much more dramatic and subversive than what was to be her last Moomin book, *Moominvalley in November*” (2014/2007, p. 294).

The scene represented by the quote is symptomatic, as it concludes Moominpappa’s long struggle with the sea and represents the much-anticipated reconciliation, which while overseen, gives us access merely to a partial message of the book, whose intriguing interpretation was put forward by Torsten Rönnerstrand in the study “Barn- och ungdomslitteraturen ur jungianskt perspektiv” (1992).

Rönnerstrand points out that *Moominpappa at Sea* can be read as a literary portrayal of the Jungian individuation process—an individual integration of the conscious and unconscious. At the outset of this sea novel, Moominpappa is at a loss, harassed by the feeling of a lack in the meaning of life, typical of the second stage of the process when individuals lose contact with the unconscious. He feels himself distinctly and painfully redundant, “everything there was to be done had already been done or was being done by somebody else. Moominpappa aimlessly pottered about in his garden, his tail dragging along the ground behind him in a melancholy way” (Jansson, Tove. (2019/1965). Moominpappa at Sea. Transl. Kingsley Hart. London: A Puffin Books. 2019/1965, p. 1). This suggestive opening scene is portrayed typically with a sense of humour generated by hyperbolizing and distinctly manifests the double address. As always, the signals of the need for change are immediately recognized by Moominmamma, who decides that the family will move to Pappa’s island, where they will “lead a wonderful life, full of troubles” (Jansson, Tove. (2019/1965). Moominpappa at Sea. Transl. Kingsley Hart. London: A Puffin Books. 2019/1965, p. 15).

When after a long night boat journey, they finally arrive at the secluded, lonely island, Pappa’s encounter with the sea—which, as Rönnerstrand argues, symbolizes the collective unconscious^4^—starts. The waters appear to be astonishingly alien, “This was the great ocean, who knows how many fathoms deep, and it was quite unlike the sea and the waves that played round the jetty back there at home” (Jansson, Tove. (2019/1965). Moominpappa at Sea. Transl. Kingsley Hart. London: A Puffin Books. 2019/1965, p. 54). As the story unfolds, Moominpappa’s attitude to the sea evolves from protecting himself and building a stone breakwater, via fighting with it while fishing during the storm, to exploring it intellectually, “my idea is to discover what secret rules the sea obeys. I shall never be happy on this island until I’ve learned to like the sea” (Jansson, Tove. (2019/1965). Moominpappa at Sea. Transl. Kingsley Hart. London: A Puffin Books. 2019/1965, p. 212). In order to solve the mystery, he employs a strictly scientific methodology: regular observations, empirical experiments, proof, arguments, chapters divided into facts and speculations… He writes a thesis, but no reasonable conclusions can be drawn, and even this method fails, “The confusing thought that the sea obeyed no rules at all returned. He tried to dismiss it from his mind quickly. He was determined to understand, to solve the mystery of the sea so that he would learn to like it and be able to keep his self-respect” (Jansson, Tove. (2019/1965). Moominpappa at Sea. Transl. Kingsley Hart. London: A Puffin Books. 2019/1965, p. 205).

Eventually, it turns out that the unconscious cannot be understood in rational terms but must be unconditionally accepted, and the last scene of the book expressed in the quote used at the beginning of #OURSEA illustrates precisely this reunion that completed Moominpappa’s quest for psychic wholeness. The sea, a symbol of the unconscious, is frequently depicted anthropomorphically as bad-tempered, hostile and mean. When applied selectively and focused on the argument that suits the campaign’s goals it reduces a pivotal point of the book and cements a single-address reading.

Admittedly, another post promoting #OURSEA is more elaborate and presents an overview of diverse sea motifs in a couple of Moomin books. Interestingly, an invitation to “a deep sea dive with some of Tove Jansson’s most beloved books as guide” (Moomin, 2020c) starts with a quote on the picturesque underwater scenery taken from *The Summer Book*—classified as literature for adults—but the other quaint landscape depicts Moomintroll’s dive from the beginning of *Comet in Moominland*, when the brave diver who dared to keep his eyes open is awarded with a fairy-tale like view, “forests of crinkly seaweed swaying gently in the current—seaweed decorated with beautiful white and pink shells” (Jansson, 2019/1946, pp. 9–10). Additionally, there is a depiction of a fantastic underwater journey in the Oshun Oxtra from *The Exploits of Moominpappa*, when the boat was surrounded by friendly, colourful fish. It is again a final scene concluding a thrilling journey in which the characters were chased by an underwater monster, “In the silent dark we heard the panting of the Sea Hound chasing us. Now his grey snout with the long drooping whiskers appeared, horribly lighted by his evil yellow eyes” (Jansson, Tove. (2019/1950). The Exploits of Moominpappa. Transl. Thomas Warbuton. London: A Puffin Books. 2019/1950, p. 132).

Beneath, in the same post, it is also stated that Jansson created a sadder version of the ocean floor, too, which is illustrated with a scene, again from *Comet in Moominland*, of the main characters, who are crossing the dried-up sea on their way back home. This motif is cleverly interwoven with the #OURSEA message, “The almost apocalyptic vision could be an accurate description of how some parts of the Baltic Sea look today, since the eutrophication has led to many regions lacking oxygen and large parts of the plant- and animal life have disappeared” (Moomin, 2020c).

In #OURSEA positive, idyllic portrayals of the sea in the Moomin books are used, with the exception of one apocalyptic vision from *Comet in Moominland* which serves as a warning. It is not an inclusive picture of the sea, whose perilous side has been omitted, whereas practically in each book, beginning with *The Moomins and the Great Flood*, it is featured verbally and visually in an ambiguous way. It is alternately a source of endless gifts, not least Moominmamma, and a life-threatening, incomprehensible element. Jansson’s sea is not homogeneous, tamed, and predictable. It has many faces, because it is alive.

## Moomintrolls, the Groke and Angry Poles

The Polish constitutional tribunal’s decision, on 22 October 2020, about tightening of the already restrictive abortion law, triggered several-month countrywide mass protests, the largest since the fall of communism in 1989. Hundreds of thousands demonstrated in the streets not only against the court, whose verdict turned into merely a flashpoint, but also against the policy of the ruling national-conservative party, Law and Justice (PiS) and the Catholic Church supporting it. The protests were organized by All-Poland Women’s Strike, a social movement fighting for women’s rights, and participants displayed their anger using various symbols which have come to signify this social revolt. Among the most popular were a red lightning bolt, a meaningful yet vulgar slogan “F…k off!” directed at PiS, and a black umbrella. Interestingly, the Finnish trolls were also implemented in the imagery of the anti-government demonstrations.

On the same day that the tribunal announced its decision, Kaja Godek, a conservative Polish anti-abortion activist, released in social media a symbolical message to her Downs syndrome son, beginning with “Wojtek, you know? It is no longer possible to kill little moomins” (Wprost, 2020). Godek’s reference to Moomins was surprising for many Poles, since it is not obvious to all in Poland that this term has been used when denoting people with disabilities by Wiara i Światło,^5^ a Polish representative of the international Christian charitable association Faith and Light. The post by Godek, who is infamous in opposition circles due to her anti-LGBT standpoint, went viral in both social and traditional, oppositional media, and its fame grew even greater when it was met by Moomin Characters Ltd’s official FB response:

For Tove Jansson, who wrote and illustrated the Moomin stories, the freedom of choice in life and art was one of the most important values that guided her art and work—including her beloved Moomin stories. We, at Moomin Characters Oy Ltd, the company Jansson founded in the 1950s, do not accept the use of Moomin stories for political purposes (Wprost, 2020).

From that point Moomins became ingrained into protesters’ imagery, representing different levels of quality: from fumbling black and white drawings on a piece of cardboard to professionally printed, colourful images. They were generally based on a popular picture of the Moomin family and friends, available on the internet, and were adorned with the slogan “F…k off!” Sometimes the characters smiled, like in the original version, while sometimes their snouts frowned, expressing anger. The message was clear-cut—even the good-hearted Moomins lost their temper in the face of the government’s disrespectful policy.^6^

The other recurringly employed figure was the Groke, portrayed either in her original shape, known in particular from cartoons, or merged with Godek’s face. In some cases, the figure was individualized with a red lightning bolt on her dark dress and an inscription, for example “Respect from Moomins!” It is noteworthy that this representative character from Jansson’s legendarium was again associated with her stereotyped role. The Groke occurs in four of nine Moomin books^7^ and exemplifies, at least initially, a source of fear. Jansson believed that a good children’s book had to include elements of fright because children enjoyed it, so she implemented it eagerly in the Moomin series.

The Groke’s first occurrence in *Finn Family Moomintroll* is preceded by the warning by the newcomers Thingummy and Bob in their own foreign language, “‘The Groke is coming!’ whispered Bob. ‘Groke? Who is that?’ asked the Hemulen, getting a bit frightened. ‘Tig and brim and gerrible!’ said Bob. ‘Lock the door against her’” (Jansson, Tove. (2019/1948). Finn Family Moomintroll. Transl. Elisabeth Portch. London: A Puffin Books. 2019/1948, p. 127). It turns out that the doors do not have any keys in the Moomin house, but the family arms itself and moves furniture to protect the entrance. When the Groke finally arrives, she merely sits motionless and stares at them: “She was not particularly big and didn’t look dangerous either, but you felt she was terribly evil and would wait for ever. And *that* was awful” (Jansson, Tove. (2019/1948). Finn Family Moomintroll. Transl. Elisabeth Portch. London: A Puffin Books. 2019/1948, p. 29). The place where she is sitting is frozen, and thus her primary quality is established—the Groke embodies cold.

In the following volume, *The Exploits of Moominpappa*, her characterization crystalises and she becomes even more gruesome. It turns out that she howls terribly and can eat up other beings, although it is expressed in a typically comical convention. When Joxter glorifies the idle lifestyle, he warns his friends of the dangers of education: “I had a cousin who studied trigonometry until his whiskers drooped, and when he had learnt it all a Groke came and ate him up”^8^ (Jansson, Tove. (2019/1950). The Exploits of Moominpappa. Transl. Thomas Warbuton. London: A Puffin Books. 2019/1950, p. 33). Surprisingly, the Groke—although the landscape fits her perfectly—merely passes several times in the background of *Moominland Midwinter* and plays a slightly bigger part in only one scene, when she sits down on a great, ritual bonfire, whose goal is to wake up the sun, and put it out. When Moomintroll worries that now the sun will stay away, Too-ticky comforts him: “She didn’t come to extinguish the fire, you see, she came to warm herself, poor creature” (Jansson, 2017/1957, p. 76). Thereby, we are acquainted with the Groke’s new trait—she is attracted to light because she is cold and lonely, and thus she deserves sympathy.

Importantly, the Groke is a character of the last book featuring the Moomin family, *Moominpappa at Sea*, although a lot of crucial and recurrent characters (i.e. Snufkin, Sniff, Fillyjonk and Hemulen) are absent. Already at the beginning Moominmamma expresses an opinion which corresponds with Too-ticky’s: “we are afraid of the Groke because she’s just cold all over. And because she doesn’t like anybody. But she’s never done any harm” (Jansson, Tove. (2019/1965). Moominpappa at Sea. Transl. Kingsley Hart. London: A Puffin Books. 2019/1965, p. 14).

However, a direct confrontation with the Groke occurs only between her and the Moomintroll, who, in this volume, grows up, loosens the bonds with his beloved mother and becomes distinctly independent. One of the elements of his maturation is thinking about the Groke and trying to understand her, “If she was someone you mustn’t talk to or about, then she would gradually vanish and not even dare to believe in her own existence” (Jansson, Tove. (2019/1965). Moominpappa at Sea. Transl. Kingsley Hart. London: A Puffin Books. 2019/1965, p. 29). Further, the plot unfolds as a several act play about “subjugation of the monster”, during which the Groke regularly comes out of the sea to stand closer and closer to the lamp, exhibited specially for her by the Moomintroll on the shore of the island. This process is reminiscent of the taming of the fox by the Little Prince in the de Saint-Exupéry classic, which consisted in—as the animal explained—establishing ties to become unique to each other, and concluded: “If you want a friend, tame me” (de Saint-Exupéry, 2017/1943, p. 70). The mechanism of making friends in *The Little Prince* involves observing at a distance and pretending not to see each other, and then sitting closer and closer every day.

Moomintroll’s relationship with the Groke is also silent and at first devoid of eye contact—words become dispensable, and the patience and curiosity of the other are of the highest significance. The Groke makes a ritual of the evening meetings, and even when paraffin is missing and the Moomin is unable to light the hurricane lamp, she comes ashore reacting to his sight:

Suddenly the Groke started to sing. Her skirts fluttered as she swayed to and fro, stamping on the sand and doing her best to show him that she was pleased to see him.

Moomintroll moved forwards in amazement. There was no doubt about it, the Groke was pleased to see him. She didn’t mind about the hurricane lamp. She was delighted that he had come to meet her.

He had stood quite still until she finished her dance. Then he watched her shuffle off down the beach and disappear. He went and felt the sand where she had stood. It wasn’t frozen hard at all but felt the same as it did (Jansson, Tove. (2019/1965). Moominpappa at Sea. Transl. Kingsley Hart. London: A Puffin Books. 2019/1965, p. 245).

Thus, the Groke, viewed via a comprehensive reading of the Moomin saga, appears as a victim of groundless ostracism, but symptomatically, the abortion strike protestors in Poland used her one-sided image of a fright-evoking figure, based on her initial characterization, reinforced by the cartoons.^9^

## Final Considerations

As I have tried to show, the presence of Moomins in societal campaigns has been discernible for over two years and thanks to largely social media, it has gained world-wide coverage. The analytical juxtaposition of relations between the images from Moominvalley employed in the three events and their literary sources suggests interesting conclusions. The motifs and characters used in these campaigns both by professionals and average readers reify a selective reading and cement the single address of the Moomin suite. The unique value of Jansson’s books is a combination of universality, diversity and avoidance of simplistic solutions. On the one hand, it is indisputably a fact that in Jansson’s saga Ninny is invisible, the sea is beautiful and the Groke is gruesome. On the other hand, the same Jansson, let us not forget, argues that the invisible can be male, the sea can be angry and the Groke can be vulnerable.

## Footnotes

1 These were: *The Exploits of Moominpappa* (1950), *Moominsummer Madness* (1954), the collection of Moomin short stories *Tales from Moominvalley* (1962), *Moominpappa at Sea* (1965), and the final book where Moomins are missing, *Moominvalley in November* (1970). Jannson also authored three brilliant picturebooks: *The Book about Moomin, Mymble and Little My* (1952), *Who Will Comfort Toffle?* (1960), and *The Dangerous Journey* (1977).

2 Snork Maiden’s characterization was met with criticism by Kirsten and W Glyn Jones, which Boel Westin mentions in her book *Familjen i dalen* (1988, p. 159). These critical arguments seem to be removed from the discourse around Jansson and are now replaced with emphasis on her feminism.

3 I am discussing here this species collectively, although there are different representatives of fillyjonks in the series.

4 Rönnerstrand’s (1992) argument encompasses other parallels to the individuation. First, he highlights the connection between a small lake, discovered by Moominpappa and called the black pool, representing the individual unconscious, and the sea—the collective unconscious. Second, the scholar explores a motif of the lighthouse lamp that does not work until the ending of the book. Its lighting enhances a process of psychic wholeness in symbolical terms, as the round shape of the lighthouse tower stands for a mandala, a representation of the integrated self.

5 There are two other names of the association in Poland: *Muminki* (Moomins) and *Wspólnoty muminkowe* (Moomin communities). Within the community, intellectually disabled children have been called Moons and their caretakers - Hemulens. The reference to Moomins was originally supposed to relate to the atmosphere of an ideal family. Over time, however, the division into two opposing groups began to arouse dissatisfaction of both children and their parents (Pessel, 2013, p. 312).

6 One of the popular images represented the angry Moominmamma with red lightning on her snout. I see it as a kind of provocation expressing the message: Moominmamma, who embodies perfect kindness, a perfect mother and wife, got really mad at the women’s situation in Poland and joined the strikes.

7 They are *Finn Family Moomintroll* (1948), *The Exploits of Moominpappa* (1950), *Moominland Midwinter* (1957)??, and *Moominpappa at Sea* (1965).

8 Grokes, like for example fillyjonks, represent a species. If they stand for one specific character, they are described in the definite form. Sometimes Jansson amplified this distinction using either small (for a species) or capital (for one representative) letters, but she was not consistent. In the Polish translation the distinction between ‘a Groke’ and ‘the Groke’ disappears as they are rendered as one particular character, and consequently viewed in this way by readers.

9 It is noteworthy that the strike protestors were predominantly the Millennials who were acquainted with Moomins through Japanese cartoons whose plot rested on the singe-address of the books, targeted at children. I argue the Moomin images still result from a selective reading of the mediators, the cartoon makers.
